# *Organon* § 16

**Published:** 1892-10

**Authors:** Arthur G. Allan

**Affiliations:** Philadelphia, PA


### *ORGANON*, § 16.

ARTHUR G. ALLAN, M. D., PHILADELPHIA, PA.

In the sixteenth paragraph of *The Organon* we find the following words:

"Our vital-force being a dynamic power can be affected only in a purely dynamic manner by the noxious influence of the hostile agents acting upon the healthy organism which come from without to disturb the harmonious play of life. Therefore the physician can remedy these disturbances, diseases, only by bringing to bear upon them some substance endowed with a force *equally* dynamic or virtual from which the vital-force receives the impression by the aid of the nervous sensibility everywhere present."

I have chosen this section of *The Organon* as my subject because it sheds a ray of light upon a most important point—the administration of medicines. We are daily confronted with the following questions:

1st. In what potency ought the remedy to be given?

2d. In what manner ought it to be administered?

We all admit that we have an absolute rule for the selection of the remedy, and certainly there must be also just as definite a rule for its administration as there is for its selection. It is a great thing to know how to select the remedy, but it is equally as important, if not more so, to know how to give it, since of what value is something the use of which is unknown? Can a man who has a large assortment of surgical instruments become a surgeon without knowing how to use these instruments? It is not the instruments or the knowledge of what they are for that makes a surgeon skillful, but it is the knowledge of how to use these instruments. In like manner it is the knowledge of how to give the remedies after they have been selected that will make a physician skillful in the healing art, and not merely a knowledge of how to choose remedies under the law, *similia similibus curantur*. It is not sufficient to say that too much of the remedy must not be given and that we must not repeat doses too often, but give a single dose of the remedy and then let it have plenty of time to act; but we must know how the remedy acts and what part it plays in effecting a cure in order that we may be able to give definite reasons for this assertion. The human mind is not satisfied to know a bare fact. In its thirst for knowledge it craves for the *reason why*, and unless this desire is satisfied it fails to appreciate and retain even a most valuable truth.

In considering the meaning of Hahnemann's words when he says that, since diseases are dynamic in their origin, they must be cured by powers that are *equally dynamic*, we are impressed at once with the idea that in order to obtain medicinal powers that will be of an equal dynamization to the disease power, medicines must be potentized.

This is so evident that there can be no other conclusion formed. The vital-force being an invisible power and the disease itself being likewise an invisible power, it follows that to cure disease we require as a medicine another equally invisible power. It is quality, not quantity, that is necessary to work a cure. Therefore, potentization of medicines is essential to Homœopathy; one cannot practice it unless he employs medicines in this manner.

But it is not sufficient to stop here.

Hahnemann has said that as the disease is dynamic the medicine must be equally dynamic, and this brings us to another important consideration. The medicine should have dynamic properties equal to the disease. Now, do all diseases have the same degree of dynamization, or is there any variation in them, and, if there is, what is it and how can it be determined? If we observe two patients suffering, one from an acute disease, and the other from a chronic one, it will not take long to see that the two diseases are unlike in action. The acute disease develops suddenly and has violent symptoms, indicating a great intensity of action. On the other hand, the chronic disease develops slowly, almost imperceptibly, yet with irresistible power, and finally overwhelms the vital-force; in action the opposite of the acute disease. These two diseases, the acute and the chronic, may both require the same medicine to bring about a cure, and yet, as we have just remarked, in intensity and mode of action they are wholly unlike. Consequently the medicine (potency) that is similar in action to the one case will be wholly unfit for the other. To suppose that we can employ one set of potencies for all diseases is as grave an error as alternation, etc. The action of a potency is not like that of the crude drug. Many medicines that are active in potency are apparently inactive in the crude form. So with the various potencies—the low, the medium, and the very high ones—vary in their action. In studying the action of a medicine in its various potencies it will be found that the low potency has a much more prolonged and much less intense primary action than a high one. The high potencies impress the vital-force suddenly and their action has a shorter duration than has that of the lower potency. The higher the potency, the greater the initial intensity and the shorter the duration of the primary action; so that in comparing the action of high and low potencies with the action of disease we notice at once that low potencies resemble chronic disease by having a much more prolonged and much less intense action, and that high potencies, by having a more rapid and intense action upon the vital-force are similar in action to acute diseases. For this reason, in order to employ medicines that are *equally* dynamic to the disease, we should use them in low potencies in chronic diseases and in the highest potencies in acute ones. In chronic diseases the highest potencies are *not similar*. On account of their great intensity of action they are more liable to aggravate than low ones, but in acute diseases the high potencies are much less likely to cause an aggravation than the low ones. The danger from the use of crude drugs and very low potencies in acute diseases is not alone from the homœopathic aggravation which medicines are likely to produce, but a very low potency, or a crude drug particularly, not being similar in action to an acute disease, in place of provoking a curative reaction may merely irritate and excite the already over-sensitive nervous state of the patient, thereby aggravating his condition without the possibility of a curative reaction taking place, notwithstanding the fact that the medicine may have been correctly chosen. Although this is an aggravation, it cannot be considered in any sense of the word a homœopathic one. Finally, there is a condition of chronic disease where the highest potencies are to be given the preference over low ones. It is in those cases where allopaths have subjected the patient to a prolonged course of treatment. In these cases it has been the custom among homœopaths to give Nux to calm the nervous excitability that such a treatment has produced. Likewise for the same reason it is impossible to derive any benefit from low potencies until the high ones have quieted the patient and antidoted the effects of the previous drugging.

Having seen that we are to begin the treatment of *acute diseases* with *high potencies*, and *chronic diseases* with *low ones*, we now come to another important subject:

When is it best to give a medicine in repeated or broken doses?

How many of us are there who have not at times been perplexed and unable to decide whether to give a single dose and wait, or to give a succession of doses? The remedy has been selected, but how shall it be given? This is a question that has disturbed us all, although there is just as definite a rule for the administration of doses as there is for the selection of the remedy itself. To understand this subject we must continually bear in mind that every medicine when administered produces two effects—a primary or direct action, and a secondary action or reaction, which is the curative one. If, after a single dose of medicine has been administered, another one is given before the primary action of the first dose has ceased, it will cause an increase of, and a prolongation of the primary action which the first dose produced. If a third or fourth dose be given in the same manner, it will still increase and prolong this primary action, each succeeding dose intensifying to a certain extent the primary effect of the preceding one. The object of the administration of medicines being to produce a reaction that will blot out the disease, let us see what effect such a succession of doses will have upon the reactive or curative action. The reaction after the administration of a medicinal force in disease depends for its intensity and duration upon two circumstances—the length of time that the primary action lasted, and upon the amount of force that the medicine exerted over and above that of the disease. We have already seen that the effect of a succession of doses is to increase the intensity of the primary impression of the first dose, and also to prolong the primary action, so that we must observe that the only time when a succession of doses is admissible is when we find the single dose fails to produce a primary impression upon the vital-force of sufficient duration and intensity. The cases in which such a procedure is advisable are those in which the disease action is very intense, and where a single dose would have but a slightly greater intensity of action than the disease, and be followed by so short a reaction that it would accomplish very little before its effects would be lost altogether. "The medicine," said Hahnemann, "must be more intense in its action to supplant the disease than that of the disease force," and it is to insure this greater intensity of action that we are to give divided doses. In acute diseases alone do we find an intensity of disease action which requires so very great an intensity in the medicinal action to supplant it that it is advisable to resort to broken doses.

In chronic disease the opposite exists. The action of the disease force in chronic disease is of so very little energy that a single dose will have an intensity of action so much greater than the disease that even a single dose of the highest potencies will often produce, in susceptible persons particularly, a dangerous aggravation, and at times even "spoil the case," as we call it. Thus, we observe *that broken doses are only suitable for acute diseases, and they never should be given in chronic disease under any circumstance whatever*.

Having now found out what kind of disease requires broken doses there arises right here another question: How long can this repetition of the medicine be continued with good results? This is a question which experience alone can determine and must be left to the judgment of the prescriber. One case may require a more frequent repetition than another, so that no arbitrary rule can be laid down for every case ; but my own experience in the matter leads me to consider that it is not advisable to continue this repetition more than six hours, and usually six doses at intervals of half an hour are amply sufficient.

It often happens that after the lapse of twelve or twenty-four hours we find that some of the symptoms remain or we notice that the disease is again increasing, making it advisable to repeat the remedy. Then the question at once arises : Will it be better to repeat the remedy in broken doses or give it in a single dose? Here again we must be guided by what we are endeavoring to do with the remedy which we are administering, and also what we have already accomplished by the broken dose which has been given. Bearing in mind that it is the reaction of the vital-force that cures and not the primary action of the medicinal force, we must now give only enough to stimulate the vital-force to react. The effect of the broken dose already given has been to diminish the intensity of the disease action ; but a cessation of the amelioration which the broken dose had started or the increase of the disease again is an evidence that the dose has failed to produce a sufficiently powerful and prolonged reaction. Now, the necessity in the case is to continue the reaction which the first dose had started. The acuteness of the attack having been modified by the first dose, we no longer find it necessary to give broken doses to insure a high degree of intensity of action as in the administration of the first dose ; but it is merely necessary to give encouragement, so to speak, to the vital-force to continue to react. For this purpose a single dose alone should be given. In fact, the administration of broken doses again would subject the patient to the dangers of an aggravation or what might be called a proving of the remedy. And this brings us to a point that is quite *apropos* to the occasion : Is it best in repeating to give the remedy in the same potency or in a different one, and if a different one, should it be a higher or a lower one ? The first dose was given in the highest potency because the highest potencies resemble acute disease in action ; that is to say, in intensity of action. The medicine must have greater intensity of action than the disease in order to supplant it and produce an impression upon the vital-force. That it did have this is manifested by the fact that it modified to a certain extent the violence of the attack. But the second dose which we are now called upon to administer is given, not to obtain an intense action which the disease modified by the former dose does not now require, but to produce a prolonged reaction which the first dose even in broken doses failed to do. As we have seen above, lower potencies produce a more prolonged reaction than the higher ones, it follows that a lower potency will correspond better to the present condition than the higher one, and, consequently, when repeating the dose in acute disease we had better go lower than higher. Experience has proved to my perfect satisfaction this deduction to be correct. But while I say in repeating in acute disease give a lower potency, I wish to make clear the point that it is to be presumed that an amelioration has already taken place from the previous dose, and that the disease has now come to a standstill or is beginning to increase again; because had there been no amelioration from the previous dose it would be necessary now to give a higher potency as corresponding to the acuteness of the attack. Consequently we must not, in repeating, give a lower potency unless a marked amelioration has taken place from the previous dose.

We have seen that broken doses are only suitable for acute diseases; but are there cases of acute disease where broken doses would not be appropriate, or where it would be better to give a single dose and await the result before repeating? We have seen that broken doses produce a very powerful primary impression upon the vital-force, and if for any reason there be any objection to producing such an effect, then it would not be advisable to give broken doses of the medicine. Should we in any case find the vital-force weakened, then so powerful an impression as broken doses would produce might so overwhelm the vital-force that a curative reaction would be impossible. Again, if the disease be one that has a definite course to run, since the medicine would not be able to arrest the already developed disease, it would be bad policy to give more than a single dose at a time. In looking over the patients and diseases that come under our charge we notice that old people have much less vitality and power to react than young ones. In them, especially the very aged, broken doses will do more harm than good. Age has so weakened their power to react that not alone are broken doses not suitable for them, but they may, from the intensity of their action, prove injurious. For the same reason it is even dangerous in some of these cases to administer the very highest potencies even in a single dose, lest the primary impression of the medicine be of too great an intensity and a curative reaction fail to follow.

Again, there are diseases which affect so powerfully the blood and the tissues of the body, breaking them down, that in these it would likewise be bad policy to administer broken doses of the highest potencies. For example, such a disease is diphtheria. In this disease so powerfully is the system affected and so broken down does the blood become, that the reactive power of the vital-force is so weakened that it is really dangerous to repeat the dose under at least ten or twelve hours. In such cases it is far preferable to give a single dose and wait than, in the anxiety to be doing something to hasten the cure, to be actually endangering the life of the patient.

In zymotic disease and all diseases that have a definite course to run, it is never proper to give divided doses. In the first place, the object of the treatment is to diminish the intensity of the disease and thus render it harmless so that it can run its course without endangering the life of the patient. A single dose of the homœopathic remedy will do this by causing a reaction that will modify to a considerable degree the severity of the attack without preventing the disease from running its course. But could we set up such a powerful action from our medicines as would supplant and overshadow the disease, we should gain nothing so far as the good of the patient would be concerned. Either the disease would be suppressed by the medicine, which is most dangerous for the patient, or the whole case would be aggravated, to his great risk. We must always remember that, in eruptive fevers in particular, we ought not to attempt, after the disease has become fully developed, to cure, shorten the duration of the disease, or interfere with the development of the eruption. In these cases medicines must be given in single doses at long intervals. At least twelve or twenty-four hours should elapse between doses, and, if every symptom has been modified by the remedy, it is much better to wait even a longer time than that before repeating. Nothing will ever be gained by impatience. In cases of this nature it is far better to wait and be sure, than to do harm by unnecessary interference.

And now, in closing, allow me to say that while this paper is not intended to give all there is to say upon an important subject like this, enough, it is hoped, has been said to show the principles upon which remedies are given and to assist others in prescribing and in conducting future observations.

### Discussion.

Dr. A. R. Morgan—What distinction does the gentleman make between divided doses and repetition?

Dr. Allan—There is a real distinction between divided doses and repetition that may be plainly seen. To put some medicine in a glass, and give a teaspoonful every half hour for a few hours until improvement is manifest, is simply a divided dose. To give a patient a bottle of medicine, and tell him to take a dose every few hours or three times a day for an indefinite length of time is repetition. I do not mean the latter at all.

Dr. A. R. Morgan—The gentleman makes a distinction on the subject of dose with which I do not agree. I regard a dose every few hours as a repetition.

Dr. Wm. Wesselhœft—This is a matter of personal experience largely. As I understand Dr. Allan, he claims that it is better to give a high potency in a broken dose in highly acute diseases, in order to make the first impression, while in chronic diseases it is better to give only the single dose to make the first impression.

Dr. A. G. Allan—In answer to Dr. Morgan, I want to say that some doctors give a single dose and await results, others give four or five doses in water or powder until some improvement is manifest, and call it a broken or divided dose, this latter is a very different thing from the indiscriminate repetition practiced by many.

Dr. Kimball—Dr. Gregg, in his work on diphtheria, recommends that the second dose of a remedy in diphtheria should never be given, if you have a good effect from the first dose, without waiting twelve or twenty hours, and that more than two doses of the same remedy should not be given without waiting several days, or giving a dose of another remedy.

This does not seem to agree with Dr. Allan's advice to give broken doses in acute diseases.

Dr. A. G. Allan—I have found it is a dangerous thing to give a succession of doses in diphtheria. The patient is generally in a weakened and broken-down condition, and the aggravation which may take place from the remedy is a dangerous thing. It is better to give a single dose, and then wait from twelve to twenty-four hours before repeating, in the meantime giving the patient water or sugar to satisfy his mind.

Dr. W. L. Reed—I think it is a fatal error to repeat the dose, too soon, either in acute or chronic cases. If Homœopathy is a law, then disease can be cured by one dose of a medicine, and not by repetition. No repetition till first dose has become exhausted in the organism. Where the remedy is homœopathic to the case I have best results from only one dose.

Dr. T. S. Hoyne—I agree, in the main, with Dr. Reed, but there is one point he has overlooked. If his single dose does not show an effect in an acute disease—take croup, for instance—in half an hour, that dose should be repeated, if he is sure he has selected the right remedy, or, if not, another remedy must be given. I have never seen a case of croup in which improvement was not apparent in half an hour or less if the remedy was correct. To give such a rule as Dr. Reed has laid down without reservation is all wrong.

Last winter we had a considerable number of typhoid fever cases, and the majority of them were characterized by hemorrhage of the bowels. In most cases Phosphorus or Nitric acid was indicated, and, as a rule, one dose would arrest the hemorrhage at once. I had occasion to see a case in consultation with Dr. Bacon, of Englewood, in which the patient was having stools every five minutes of bright red blood, profuse as if an artery had been cut. Both Phosphorus and Nitric acid had been given when I saw the case, and injections of Hamamelis and hot compresses had also been used without effect. I found the patient almost pulseless, with slight nausea and great pallor. I gave a dose of Ipecac., and waited half an hour. Now, if I had followed Dr. Reed's rule, the patient, I am sure, would have died. But I gave a dose of Secale-cornutum, which controlled the hemorrhage almost immediately, and after a long illness the patient recovered. The patient had no evacuations for forty-eight hours afterward, then they were black and tarry. None of the books I consulted gave Secale as a remedy for typhoid, except P. P. Wells', and he does not mention hemorrhage as a symptom.

Dr. A. Campbell—Did I understand aright that Dr. Allan said we are never to undertake to abort or arrest the progress of an eruptive disease?

Dr. A. G. Allan—That is what I said. You must not expect to arrest or stop an eruptive disease after the development of the rash. If you give many doses of the remedy in such cases, you will not have as good a result as if you give a single dose and wait.

Dr. Campbell—If you cannot hope to arrest an eruptive disease, why give any medicine at all?

Dr. A. G. Allan—To modify its course and prevent it from getting worse.

Dr. Campbell—Dr. Wells was practicing during an epidemic of small-pox. He told me that in a case so covered with the eruption that the tip of the finger could not find a place on the patient's body uncovered with it, he arrested and aborted the attack with a single dose of Sulphur.

Dr. G. H. Clark—I think Dr. Campbell's point well taken. No disease has a definite course under homœopathic treatment. That is what the allopaths say; we, as homœopathists ought to know better. The gentleman will find after he has had more experience in Homœopathy that he will have much to unlearn about the course of various affections.

Dr. A. G. Allan—A disease like pneumonia must take a definite course.

Dr. G. H. Clark—It must not and does not if the case is treated as it should be; homœopathically. To teach that there is a definite course in any disease is a fallacy. It is an inheritance from allopathy.

Dr. Wm. Wesselhœft—I do not think that pneumonia need have a definite course under homœopathic treatment, because even under allopathic treatment it does not invariably have a *definite* course. Sometimes resolution occurs without expectoration. We know of cases without either cough or expectoration, and then when we talk of definite stages of a disease I believe it to be all nonsense. We should have no cut-and-dried stages in diseases. It is an idea that leads a great many of our young men astray. You hear such directions as giving Aconite in the first stage, then wait for the second stage and give Byronia or Tartar-emetic, then wait for the third stage and give Sulphur. Instead of prescribing according to the symptoms of the patient, they prescribe for the stages. I had a horrible case of double pneumonia in my own family, where the patient could not be moved the eighth of an inch, where she wanted somebody to stand over her and press upon her with might and main. She got well under Arnica. She had neither cough nor expectoration.

Dr. Johnson—The question that seems to be before us is, "How long shall we wait before giving the second dose," whether we call it a broken dose, or a number of doses? We find in *The Organon* clearly taught, that all sensations, and all symptoms of disease are produced by the *vital-force.* Abnormal sensations or symptoms show that the vital-force is in an abnormal condition, and the only thing we can do is to right the wrong. If we give the *proper* remedy then the *vital-force* will correct the symptoms. This is the standard of the true Hahnemannian. If we give a dose of medicine, it is because we see in the symptoms of the patient a picture of the symptoms produced by that drug, in the proving on the healthy ; and if a change in consequence occurs it is a proof positive that the vital-force is acting. Now shall we step in and interfere with that curative action? Certainly not. The better thing to do is to wait. How long? Not two hours. Not two days. Not a week nor a month—no definite time, but wait until we see the returning symptoms, or a stop in the curing process. Then, if *indicated*, repeat the *remedy*, whether it is two hours or five months. If we see no reason for giving a remedy it is a great deal better to give nothing. Let the *vital-force* do the guessing.

Dr. T. S. Hoyne—Mr. President, Dr. Johnson misses the question entirely. How long are we to wait for a remedy to act? Suppose a case of croup. He has given a remedy and no effect is apparent. Now, how long is he going to wait on that one dose?

Dr. Johnson—Homœopathic physicians are not infallible, they do sometimes make mistakes. It is supposed they know the signs of the action of a remedy, and the signs of its failure to act. If we get no curative effect in a reasonable time from the remedy, we should choose another without delay. Why repeat a remedy that does not act, or wait too long on one that produces no effect?

Dr. J. B. G. Custis—After all we are practical men and we meet disease in a practical manner. It would be impossible for most of us to give a dose of medicine and then spend time enough to see it work. Life is too short and there are other sick people claiming our atttention. The repetition of the dose certainly does not depend upon whether the disease is acute or chronic. It depends upon the patient's susceptibility to the remedy more than anything else, and that varies with different people. Hence it won't do to say that you must wait any definite time. You must be guided by circumstances. The success of a homœopathic physician depends upon his ability to forget the names of diseases. Our best cures are often made by remedies that we have never before applied to the disease in hand. Whether the disease be eruptive or organic makes no difference at all in the selection of a remedy. The broken dose that Dr.

Allan speaks of is certainly a repetition, a number of doses. The quantity has nothing to do with it.

Another thing, I do not believe we ever set up a new diseased condition ; we cure diseases, we do not make new ones. Among every doctor's patients are people of judgment, who can be trusted to make accurate observations ; who will say, "I felt better for awhile after the first dose then the other powders did not seem to help me." With such people it is safe to give a number of powders of the medicine and tell them to stop when improvement begins. But the majority of our patients are not to be trusted in that way. However, it is sometimes impossible for us to see a patient several times in a day or two. In additon to this, I have found in my experience that some people bear repetition better than others, all of which goes to show that this is a question of experience with individual patients and circumstances and no one should presume to say how others should do or make a hard-and-fast rule for all to follow.

Dr. Baylies—Dr. Allan made an important statement in his paper which may have been misapprehended. I understood him to say that in acute cases the higher potencies should be applied, in chronic cases the lower, a method quite contrary I think to that usually adopted, and implying a view of the relative efficiency of the high and low potencies in these two grades of diseases not generally accepted.

Dr. A. Campbell—This reverses the order of things in which we have been instructed for a good many years.

Dr. A. R. Morgan—The position that low potencies are better adapted to chronic complaints and the high potencies to those which are acute is so at variance with my experience that I should like to hear the subject further discussed by the members.

Dr. S. Long—By low potencies Dr. Allan means the 200th and 1000th, nothing lower.

Dr. A. R. Morgan—In chronic cases my best results have been obtained from the highest potencies, while in acute cases I am more inclined to give the 200th.

Dr. Long—This question resolves itself into this : Press the button and we do the rest. Get your remedy and you certainly press the button, the rest follows by experience. No matter how long you discuss this question, you will still be at sea on account of the main thing being individual and not general. Before I can decide the question of repetition I want to know whether I am treating John Jones or Jane Smith. The individual and the remedy must both be considered. We all know that our remedies differ in individuality as well as our patients, and if we endeavor to so argue the matter a week we could not arrive at a definite conclusion, because it is a thing that admits of only the general law, never repeat as long as improvement continues, but not of any laws as regards details. These vary with each case.

Dr. Farley—Do the highest potencies cause a reaction that is longer-lasting as a rule, or on an average, than the lower, or is the opposite true? If we could decide that question it would go far toward deciding which cases were most apt to be benefited by the high and which by the low potencies.

Dr. Fincke—Dr. Jaeger answers that question to a certain extent. His claim is based upon experiments that prove that the reaction produced by the highest potencies is longer-lasting and more powerful.

Dr. McLaren—No more useful paper or discussion could have been brought before this Association. The questions involved are questions that must be solved, although they cannot be solved to-day perhaps. Dr. Allan will find in time that the last word has not been said upon the subject. There is no one here to-day too old or too young to learn about these unknown depths of Homœopathy and of the individuality of patients.

Dr. Tompkins—If I heard the paper read by Dr. Allan aright, he says that high potencies produce a more prompt and intense reaction than lower potencies, and are therefore more homœopathic to acute diseases than the lower potencies, while, for a converse reason, the lower potencies are better adapted to chronic diseases. I do not rise so much to dispute this statement, opposed as it seems to me to be to the teachings of Hahnemann and the general experience of our school, as to make this an occasion for emphasizing a needful discrimination. There are things upon which I suppose this Association is substantially agreed as to their truth; among these the persistence of curative power in the highest potencies, the superiority of the homœopathically indicated remedy over all other medication for the cure of dynamic disease, and that certainty of cure is proportioned to the exactness with which disease symptoms are matched by the symptoms of the remedy.

There are other points which even Hahnemann's great dictum has not settled for all of us, and these it behooves us to recognize as hardly capable of settlement by the experience of any single practitioner. Eminently in this list appear the questions of the appropriate potency for a given case of disease or class of diseases, and the sufficient repetition of the dose, the latter question often appearing disguised as the question of the "division of the dose." If any man's experience appears to teach something concerning either of these points he can easily put us in his debt by citing the facts; but is he justified who without preface or explanation assumes a settlement of one of them as a point of departure for an essay or argument? Again, if we should be blessed with the presence among us of some who have not long pursued the study of pure Homœopathy, are we not in danger of confusing such by assumptions of settlement of these questions which also fail to agree with the founder of our school?

Dr. Johnson—I regard the paper as important. In our clinic we make about six hundred prescriptions a month, and we are trying to arrive at some conclusion in this matter.

In chronic cases we usually begin the treatment with the medium potencies about 40 M or 50 M. If it becomes necessary in the case to administer again the same medicine, we usually give it in a higher potency. We have found by experience that if the same potency of the same remedy is again given, it does not hold the patient in an improving condition so long as the first, and if given again, the time of improvement is still shorter. We seldom repeat a remedy in the same potency more than once. It seems to become necessary to give the CM or a potency higher than the first one given. Each higher potency seems to go deeper into the life and take up the case after the lower potency has failed to produce further effect, and usually it carries the patient further toward health than the previous dose. We may have to go to the MM, or even higher, before a cure is effected. The question has been raised whether it would not be better to give the CM or higher potency in the beginning, but we have no facts that will enable us to answer that question. In acute disease we begin with the CM, and one dose is usually sufficient.

Another experience is that we have noticed a patient under treatment for chronic disease may have an acute attack, and while under the influence of the chronic remedy—Sulph., Lachesis, or Lycopodium for instance, we may give the indicated remedy for the acute attack, in a lower potency, say the 1M or 10M without apparently disturbing the action of the higher potency of the chronic remedy.

This happens occasionally only, and we would not interfere or give another remedy unless the acute disease is very severe.

Dr. A. G. Allan—It would be the height of folly if I offered this paper as settling the whole matter. I had no such intention, although some seemed to have so understood me. It is only my experience which I offer as a contribution upon this subject. I give it as a suggestion looking toward the determination of a much vexed question, nothing else—certainly not regarding it as the conclusion of the whole matter.

I maintain that the broken dose is nothing but a single dose given in division. The effect is the same as a single dose but more powerful.

By low potencies, I mean the 30th, 200th, or 1000th, but always the potentized remedy, because, if you remember, I said in my paper that it was my belief that without potentized remedies no one could practice Homœopathy.

I did not mean to touch upon the repetition of the dose at all. It is a separate subject from the broken dose. When I said twenty-four hours must intervene between doses I meant I had never seen a case in which it was necessary to give a second dose under that time, and that it might even be dangerous to do so. I have been able to observe in some cases of iritis that the action of the dose lasted for ten hours. Now, if after I had observed improvement to have fairly set in, I had given another teaspoonful or powder of the medicine, that would have been repetition and might have interfered with the cure.

Dr. J. R. Haynes—If I gather the gist of what has been said correctly, I do not agree with it, nor does it agree with the motto which we have adopted, "*Simplex, Simile, Minimum.*" I have found by sad experience that the closer I follow that motto the better my success. I think if you select the remedy aright you will seldom have occasion to repeat the dose. You do not study *The Organon* enough; if you had you would not make such speeches as you have here to-day. There has scarcely been a day, for forty years, that I have not studied *The Organon.* Years ago, like many others, I repeated the dose, but in the use of high potencies I had to quit it, and I think one of the great mistakes of the profession to-day is this matter of repetition. Get the right drug and you will not have occasion to repeat it. It will carry your case either to health or to where you will find another remedy indicated. This is my experience, and I have learned it by hard knocks.

Dr. A. G. Allan—In the epidemic of influenza which raged last winter I noticed many times that if I gave a dose of medicine, divided or broken, that the patient suffered less and got well quicker than when I gave a single dose undivided. If I gave the single dose I would probably be sent for again, but not so when I left the dose divided. We cannot be nurses as well as physicians; we cannot be at the patient's side all the time, so that the effect or action of the single undivided dose may be used up before we can get back, and the patient be worse again. So you will save yourself trouble and the patient needless suffering by leaving a divided dose to be taken for an hour or two until improvement is manifest.

Adjourned to ten A. M.

